# From plot to region: canopy height-mediated mangrove aboveground biomass mapping by synergizing UAV-LiDAR and multi-source satellite data

**DOI:** 10.3389/fpls.2026.1964480

**Published:** 2026-10-09

**Authors:** Yunyan Xiang, Wen H. Cai, Hongxin Su, Jian Yang, Le Yu, Jiali Wang, Anshu Huang, Dewen Tong

**Affiliations:** 1 Key Laboratory of Environment Change and Resources Use in Beibu Gulf, Nanning Normal University, Nanning, China; 2 School of Geography Science and Planning, Nanning Normal University, Nanning, China; 3 Guangxi Beihai Wetland Ecosystem Observation and Research Station, Beihai, China; 4 Department of Forestry and Natural Resources, TP Cooper Building, University of Kentucky, Lexington, KY, United States; 5 Department of Earth System Science, Tsinghua University, Beijing, China; 6 Guangxi Forest Resources and Environment Monitoring Center, Nanning, China

**Keywords:** aboveground biomass estimation, blue carbon, hierarchical upscaling, mangrove, structural-spectral synthesis

## Abstract

**Introduction:**

Mangroves are critical coastal blue carbon ecosystems, yet regional-scale mapping of aboveground biomass (AGB) remains challenging because of the limited availability of field measurements.

**Methods:**

To address this limitation, we developed a hierarchical structural-spectral upscaling framework in the Guangxi Shankou National Mangrove Nature Reserve, China. UAV-LiDAR-derived canopy height was first used as a structural reference to train three machine-learning models for regional canopy height retrieval using Sentinel-1, Sentinel-2, and SDGSAT-1 observations. XGBoost achieved the best performance on the held-out test set (R^2^ = 0.80, RMSE = 0.81 m) and was subsequently used to generate a continuous 10-m canopy height product. Satellite-retrieved canopy height was then integrated with multispectral vegetation indices to develop a structural-spectral log-linear AGB model based on 32 field plots.

**Results:**

Leave-one-out cross-validation showed that canopy height provided the dominant predictive contribution (R^2^ = 0.63, RMSE = 20.23 Mg ha^-1^), while the addition of the mean enhanced vegetation index (EVI) further improved performance to R^2^ = 0.68 and RMSE = 18.72 Mg ha^-1^, outperforming both strucutural-only and spectral-only models. Regional mapping estimated total mangrove AGB at 44,112 Mg, with a mean density of 49.20 Mg ha^-1^. Higher biomass was concentrated in interior stands, whereas lower biomass occurred predominantly along seaward edges and marginal zones. Biomass trajectories also differed among geomorphic settings, with increasing, decreasing, and unimodal patterns observed along different coastal-distance gradients.

**Discussion:**

Overall, the proposed framework provides a practical pathway for extending localized UAV-LiDAR structural information to regional mangrove AGB mapping while reducing dependence on wall-to-wall active sensing, thereby supporting regional blue carbon assessment and spatially targeted conservation and management of climate-vulnerable mangrove ecosystems.

## 1 Introduction

Mangroves are critical coastal blue carbon ecosystems, with exceptionally high carbon storage capacity (Choudhary et al., 2024; Dewiyanti et al., 2026). Aboveground biomass (AGB) constitutes a key component for quantifying vegetation carbon stocks and evaluating ecosystem functions (Ávila-Acosta et al., 2024; Peng et al., 2025). Spatially explicit AGB mapping at regional scales is vital for precision blue carbon accounting. Traditional forestry inventories yield high-accuracy plot data through destructive sampling or allometric scaling (Komiyama et al., 2005), but deep mudflats and periodic tidal inundation render large-scale field monitoring impractical (Chen et al., 2023a), impeding the scaling of plot measurements to continuous regional estimates. This scaling gap between sparse plot-level observations and regional management needs calls for advanced geospatial approaches that can characterize forest structure across extensive and inaccessible intertidal zones.

Satellite remote sensing has emerged as one of the most viable solutions currently available for large-scale and continuous monitoring of mangrove ecosystems (Pham et al., 2019a; Malerba et al., 2023). Passive optical sensors, such as Landsat and Sentinel-2, have been extensively employed to estimate forest structural attributes from spectral vegetation indices (Monsef and Smith, 2017; Zhen et al., 2021; Chen et al., 2023c). However, these spectral approaches are subject to a “spectral saturation” bottleneck in high-density mangrove communities (Lu, 2005, 2006). Once canopy closure approaches saturation, further biomass accumulation may produce only limited changes in top-of-canopy spectral reflectance, reducing the sensitivity of optical observations to biomass variation (Steininger, 2000; Liu et al., 2025). Active remote sensing technologies, such as Synthetic Aperture Radar (SAR), have been integrated into biomass modeling as an alternative (Hamdan et al., 2014). L-band or C-band SAR can partially penetrate the forest canopy and exhibit sensitivity to the water content of woody vegetation (Shi et al., 2023; Ullah et al., 2025). SAR nonetheless remains susceptible to saturation in high-biomass zones and interference from underlying mudflats and dynamic tidal waters (Pham et al., 2019b; Luo et al., 2024). Conventional regional mapping methods often rely on direct empirical relationships between multi-source satellite observations and field-measured AGB (Pham et al., 2020; Huang et al., 2022). Although effective in many applications, the performance of such empirical models can be sensitive to predictor selection, sample availability, and the representativeness of field observations, which may limit their robustness when extrapolated beyond sampled conditions (Lu et al., 2016).

Unmanned aerial vehicle-based Light Detection and Ranging (UAV-LiDAR) technology addresses this structural information gap by shifting from two-dimensional spectral correlation to three-dimensional canopy characterization (Wang et al., 2019; Fu et al., 2025). By emitting laser pulses capable of partially penetrating vegetation canopies, UAV-LiDAR captures the canopy geometry and underlying topography, extracting three-dimensional structural attributes including canopy height models, vertical profiles, and crown dimensions (Li et al., 2025). However, constrained by equipment costs, airspace regulations, and battery endurance, continuous full-coverage mapping across expansive landscapes remains prohibitive (Puliti et al., 2017). This operational constraint necessitates hierarchical upscaling, whereby limited UAV-LiDAR observations serve as structural baselines that can be extrapolated to regional scales using spatially continuous satellite datasets. For large-scale mapping, the integration of Sentinel-1 SAR and multispectral features from Sentinel-2 has demonstrated substantial potential (Dong et al., 2020). The Sustainable Development Science Satellite-1 (SDGSAT-1) further provides a 10-m red-edge band that is responsive to vegetation biochemical and canopy characteristics, offering additional fine-scale spectral information for characterizing fragmented intertidal landscapes (Guo et al., 2023; Ozdemir and Abdikan, 2024; Jiang et al., 2026). Previous studies have demonstrated that integrating SDGSAT-1 with SAR and UAV-LiDAR data enables accurate retrieval of mangrove canopy height (Jia et al., 2025).

Once continuous regional canopy height products are generated, a critical step in the hierarchical upscaling framework is using these structural products to derive reliable AGB estimates. Extensive research in forest ecology has established robust allometric relationships between forest structural attributes (e.g., canopy height) and biomass, providing a solid theoretical basis for incorporating remotely sensed structural variables into regional-scale AGB mapping (Hunter et al., 2013; Liu et al., 2024b). By incorporating satellite-derived canopy height as an intermediate structural variable, this framework provides a more biophysically interpretable pathway than direct spectral-to-biomass regression and facilitates spatial upscaling from localized structural observations to regional mapping (Wang et al., 2021; Yang et al., 2022). However, the potential of satellite-retrieved canopy height to serve explicitly as an intermediate structural bridge between localized UAV-LiDAR observations and regional mangrove AGB estimation remains insufficiently evaluated, particularly under limited field sampling. This approach could facilitate regional upscaling while reducing reliance on wall-to-wall active remote sensing observations.

In this study, we develop a hierarchical framework within the Shankou National Mangrove Nature Reserve in Guangxi, China, integrating field inventories, UAV-LiDAR observations, and Sentinel-1/2 and SDGSAT-1 satellite data. Our objectives are threefold: (1) to generate a continuous regional canopy-height product by upscaling localized UAV-LiDAR structural observations using multi-source satellite data; (2) to develop a structural-spectral log-linear model integrating satellite-retrieved canopy height and vegetation indices for AGB estimation, and to assess the complementary contributions of structural and spectral predictors; and (3) to map mangrove AGB at 10-m resolution and characterize its spatial heterogeneity across contrasting geomorphic settings. This study provides a practical framework for regional mangrove biomass assessment, supporting blue carbon accounting and adaptive management of vulnerable coastal ecosystems.

## 2 Materials and methods

### 2.1 Materials

#### 2.1.1 Study area

Shankou National Mangrove Nature Reserve is located in Beihai City, Guangxi, southern China (21°28′–21°37′N, 109°37′–109°47′E; **Figure 1**). The reserve encompasses both sides of the Shatian Peninsula, bordering the Xianmi Estuary and the Yingluo Bay to the east, and the Dandou Sea to the west. The region is characterized by a tropical marine monsoon climate, with a mean annual temperature of 22.9 °C (and the minimum monthly mean dropping to 16.0 °C) and a mean annual precipitation of 1573.4 mm. The tidal regime is characterized by irregular semi-diurnal tides, transitioning to localized irregular diurnal tides near the mouth of Yingluo Bay. The average tidal range is 2.53 m, with a maximum amplitude of 6.25 m.

**Figure 1:**
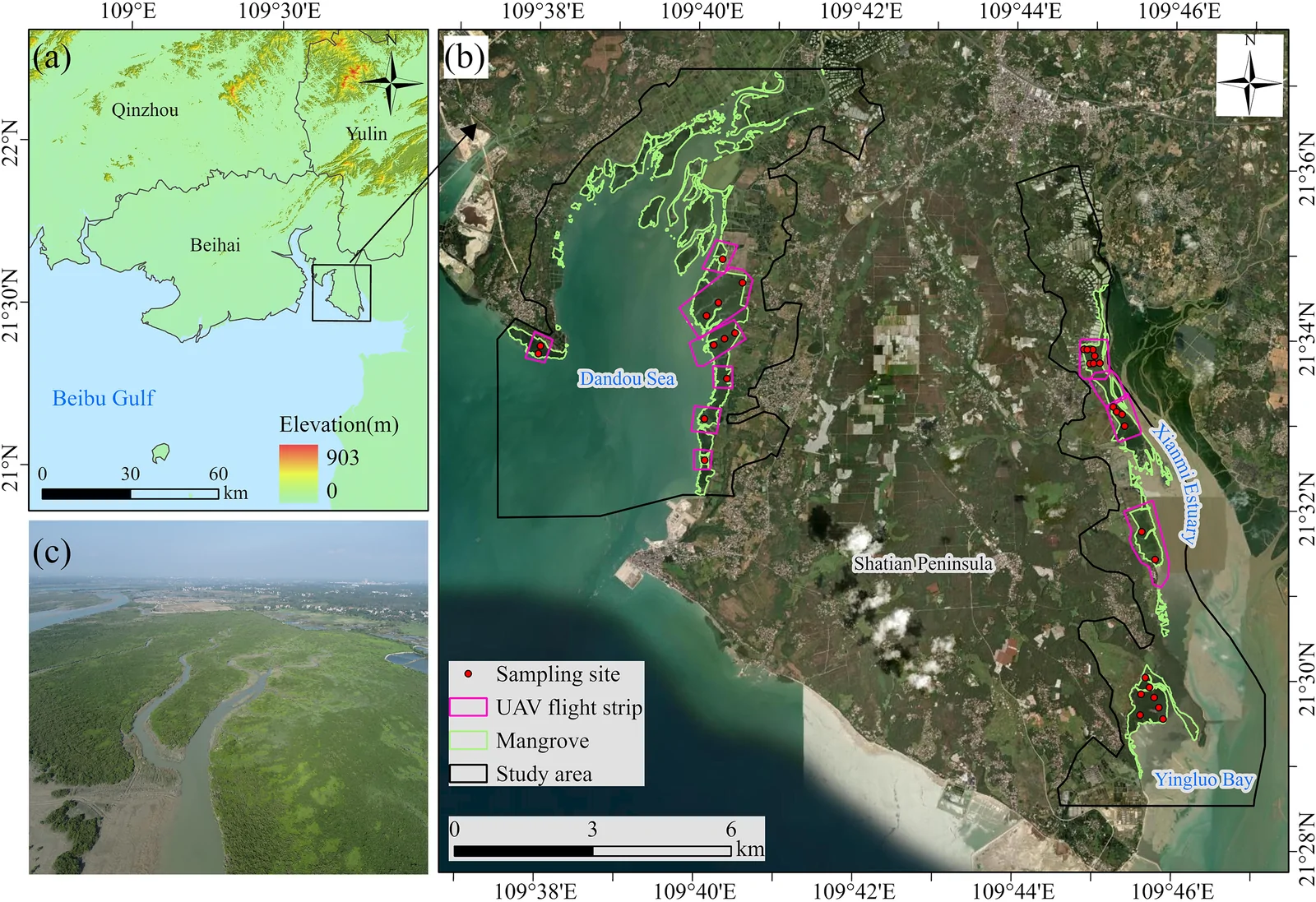
Geographic location and environmental setting of the study area. **(a)** Geographic position of the Shankou National Mangrove Nature Reserve within Beihai City, Guangxi, China; **(b)** Spatial distribution of the mangrove forests, the locations of long-term field inventory plots and unmanned aerial vehicle (UAV) flight strips within the reserve; and **(c)** An oblique optical aerial photograph captured via UAV showcasing the spatial structure and patchy configurations of the mangrove ecosystem in the Dandou Sea.

The reserve protects approximately 900 ha of natural mangrove forest, distributed primarily across the distinct habitats of the Dandou Sea, Yingluo Bay and Xianmi Estuary. In the Dandou Sea area, mangroves exhibit patchy or belt-like spatial configurations with *Avicennia marina* and *Aegiceras corniculatum* acting as the dominant species. Conversely, the mangrove communities in Yingluo Bay and Xianmi Estuary are spatially continuous and dominated by *Rhizophora stylosa*, *Bruguiera gymnorhiza*, and *Aegiceras corniculatum*. These differences in stand height, canopy closure, and community composition provide a heterogeneous setting for evaluating remote sensing-based retrievals of mangrove canopy height and AGB.

#### 2.1.2 Field survey

Field inventory was conducted in May 2023 across the Shankou National Mangrove Nature Reserve. A total of 32 sample plots (20 × 20 m) were systematically established, comprising 12 plots in the Dandou Sea area, 13 in the Xianmi Estuary, and 7 in the Yingluo Bay. Within each plot, all mangrove trees were recorded, with key biophysical parameters measured, including species composition, tree height, diameter at breast height, basal diameter, and crown width. Geographical coordinates of individual trees were acquired using a Qianxun Real-Time Kinematic (RTK) receiver, with centimeter-level accuracy. Individual tree AGB was calculated using established species-specific allometric growth equations (**Table 1**). These values were aggregated to the plot level and standardized to megagrams per hectare (Mg ha^-1^), serving as the baseline reference dataset for constructing and validating the regional mangrove AGB estimation models.

**Table 1:** Species-specific allometric equations for AGB estimation of dominant mangrove species in the study area.

Species	Component	Allometric Equation	References
*Bruguiera gymnorhiza*	Leaf	log*W_L_* = -2.217 + 1.075 log(*D*^2^*H*)	Liao et al. (1991)
	Branch	log*W_B_* = -2.219 + 1.260 log(*D*^2^*H*)	
	Stem	log*W_S_* = -1.380 + 0.969 log(*D*^2^*H*)	
*Aegiceras corniculatum*	Leaf	log*W_L_* = 0.393 + 0.475 log(*D*^2^*H*)	Tam et al. (1995)
	Branch	log*W_B_* = 1.110 + 0.463 log(*D*^2^*H*)	
	Stem	log*W_S_* = 1.198 + 0.464 log(*D*^2^*H*)	
*Kandelia obovata*	Leaf	log*W_L_* = 1.706 + 0.943 log(*D*^2^*H*)	
	Branch	log*W_B_* = 2.741 + 1.253 log(*D*^2^*H*)	
	Stem	log*W_S_* = 2.162 + 0.869 log(*D*^2^*H*)	
*Avicennia marina*	AGB	log*W* = 2.113log*D* − 0.511	Comley and McGuinness (2005)
*Rhizophora stylosa*	AGB	log*W* = 2.465log*D* − 0.696	

log denotes the base-10 logarithm. *D* represents the diameter at breast height (DBH, in cm, except in Tam et al. (1995) where it is in m); *H* denotes the tree height (m); *W_L_*, *W_B_*, and *W_S_* are the leaf, branch, and stem biomass (kg), respectively. *W* is the total aboveground biomass (AGB, kg) of a single tree. For *Bruguiera gymnorhiza*, *Aegiceras corniculatum*, and *Kandelia obovata*, *W* is calculated as the sum of *W_L_*, *W_B_*, and *W_S_*.

#### 2.1.3 UAV-LiDAR data acquisition and processing

Simultaneous acquisition of UAV-LiDAR point clouds and high-resolution visible-light imagery was conducted during low-tide windows between December 9 and 12, 2023. Flight missions were executed utilizing a DJI Matrice 300 RTK platform equipped with a Zenmuse L1 sensor. To ensure high-quality structural characterization, the flight parameters were configured with a 75% longitudinal overlap and a 50% lateral overlap, and a triple-echo mode was employed, yielding an average point density exceeding 40 points m^-2^. Post-flight processing, including trajectory reconstruction and strip alignment, was performed using DJI Terra software. Subsequent point cloud denoising, automated ground-filtering classification, and manual refinement were implemented in LiDAR360 to separate ground and non-ground returns. A Digital Elevation Model (DEM) and a Digital Surface Model (DSM) were then generated from the classified point datasets. The Canopy Height Model (CHM) was derived by subtracting the DEM from the DSM at an initial spatial resolution of 1 m. To ensure grid-to-grid alignment with the satellite imagery, the 1-m CHM was spatially aggregated and resampled to 10 m using the mean canopy height within each 10-m grid cell, providing the baseline canopy height calibration dataset for subsequent regional inversion modeling. The UAV-LiDAR-derived point cloud and a representative cross-sectional profile of mangrove vertical structure are shown in **Figure 2**.

**Figure 2:**
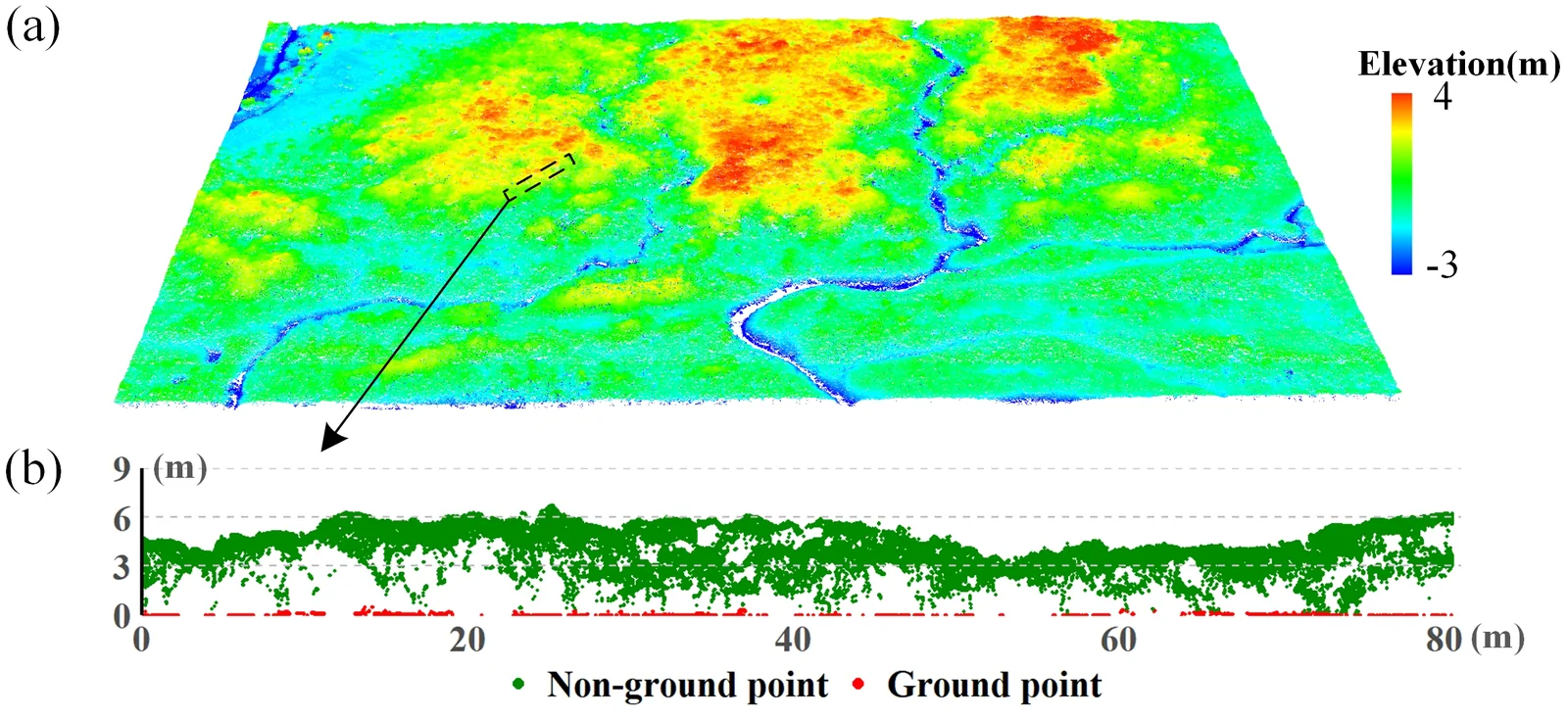
UAV-LiDAR point cloud visualization and vertical structural profile of a representative mangrove subset. **(a)** Three-dimensional perspective view of the UAV-LiDAR point cloud colored by orthometric elevation values (−3 to 4 m); **(b)** two-dimensional cross-sectional profile (0–80 m) along a representative transect, where the y-axis represents normalized height above the ground surface (m), and green and red dots represent classified non-ground vegetation returns and ground returns, respectively.

#### 2.1.4 Sentinel-1 and Sentinel-2 data acquisition and pre-processing

The Sentinel-1 and Sentinel-2 constellations, carrying C-band synthetic aperture radar and multispectral sensors, respectively, provide frequent Earth observations with an effective revisit interval of approximately 5–6 days. In this study, Sentinel-1 SAR and Sentinel-2 Level-2A imagery acquired between 25 November and 25 December 2023 were retrieved from the Google Earth Engine platform to maintain temporal consistency with the UAV-LiDAR campaign. To mitigate atmospheric and cloud contamination, cloud and cloud-shadow masking for Sentinel-2 imagery was performed using the Scene Classification Layer, followed by median compositing. For Sentinel-1, Ground Range Detected products acquired in Interferometric Wide mode were processed to extract VV and VH backscatter coefficients. Only ascending-orbit images were used, and a multi-temporal median composite was generated to reduce speckle effects before extracting the Sentinel-1 features.

#### 2.1.5 SDGSAT-1 data acquisition and pre-processing

The SDGSAT-1 Multispectral Imager provides seven spectral bands at a 10-m spatial resolution with a 300-km swath width, including a red-edge band suitable for coastal vegetation monitoring. In this study, a single orthorectified Level-4 standard product acquired on 1 November 2023 was selected because of its close temporal proximity to the UAV-LiDAR campaign and relatively low cloud contamination. The image was obtained from the International Research Center of Big Data for Sustainable Development Goals Platform. Further preprocessing focused primarily on atmospheric correction to derive surface reflectance. All satellite datasets were co-registered and resampled to a consistent 10-m grid system to match the 10-m UAV-derived CHM.

### 2.2 Methods

#### 2.2.1 Feature extraction and configuration

A total of 82 predictor variables were extracted from Sentinel-1, Sentinel-2, and SDGSAT-1 datasets to support regional canopy height modeling (**Supplementary Table 1**). For the Sentinel-1 SAR dataset, in addition to the VV and VH backscatter coefficients, three polarimetric descriptors, including the Ratio Index (RI), Normalized Difference Index (NDI), and Radar Vegetation Index (RVI), were derived to improve the characterization of vegetation structure (Jia et al., 2025). For the Sentinel-2 multispectral dataset, a suite of spectral indices was derived from the visible, red-edge, near-infrared, and shortwave-infrared bands to characterize vegetation greenness, moisture conditions, red-edge responses, and other canopy spectral properties. Among these indices, the normalized difference vegetation index (NDVI) and enhanced vegetation index (EVI) were also retained as candidate spectral predictors for subsequent AGB modeling (Munyati, 2022). In addition, GLCM texture features were derived from selected spectral bands and indices using a 7 × 7 moving window and 32 gray levels. Texture measures were calculated in four directions (0°, 45°, 90°, and 135°) and averaged, and the source bands and corresponding texture features are listed in **Supplementary Table 1**.

To retain the original spectral information, particularly the vegetation characteristics represented by red-edge bands, surface reflectance values from all seven multispectral bands of SDGSAT-1 imagery were directly used as model input features. The SDGSAT-1 spectral features and Sentinel-1/2-derived variables were integrated to construct the predictor matrix, while the 10-m UAV-LiDAR-derived CHM served as the reference canopy height product for training and evaluating the machine-learning models.

#### 2.2.2 Feature selection and multicollinearity mitigation

To reduce redundancy and multicollinearity, a two-step feature-selection procedure was applied. First, Pearson correlation analysis was conducted between each candidate variable and UAV-derived canopy height, and variables showing weak correlations (|r| < 0.10) were removed. The Variance Inflation Factor (VIF) was then calculated for the retained variables to assess multicollinearity, and variables with VIF > 10 were iteratively removed. During cross-validation, feature selection was performed independently within each training fold to prevent data leakage, and the feature selection set was then applied to the corresponding validation fold. After cross-validation, the same Pearson correlation and VIF-based feature-selection procedure was reapplied to the entire development dataset to determine the final feature set used for model retraining, independent test evaluation, and regional canopy-height mapping.

#### 2.2.3 Machine learning modeling for canopy height and performance evaluation

To characterize the nonlinear relationships between multi-source remote sensing variables and mangrove canopy structure, three machine-learning algorithms were employed for regional canopy height retrieval: Random Forest (RF), Extreme Gradient Boosting (XGBoost), and Support Vector Regression (SVR). For RF, the number of trees, maximum tree depth, and feature sampling ratio were optimized to improve model stability (Breiman, 2001). For XGBoost, the learning rate (η), maximum tree depth, subsampling ratio, and number of estimators were tuned to optimize predictive performance (Li et al., 2020). SVR was implemented using a radial basis function kernel, with the regularization parameter (C) and kernel bandwidth (γ) optimized under an ϵ-insensitive loss function (Smola and Schölkopf, 2004). Prior to SVR modeling, all predictor variables were standardized using z-score normalization, whereas RF and XGBoost were trained using the original feature values because tree-based models are insensitive to feature scaling. Grid search was used to identify the optimal hyperparameter configurations, with root mean square error (RMSE) used as the optimization criterion (**Supplementary Table S2**).

To capture spatial variability in mangrove canopy structure while limiting spatial clustering, 1,300 sample points were selected from the 10-m UAV-LiDAR-derived CHM using stratified random sampling with a minimum inter-sample distance of 20 m. The dataset was randomly divided into an 80% development subset and a 20% held-out test set (**Supplementary Figure S1**). Five-fold cross-validation was conducted within the development subset for feature selection and hyperparameter optimization. The held-out test set was excluded from all model-development procedures to prevent data leakage. Final predictive performance was evaluated on the test set using the coefficient of determination (R^2^), RMSE, and mean absolute error (MAE), calculated using Equations 1–3 as follows:

(1) $$R^{2}=1-\frac{\sum_{i=1}^{n}{\left(y_{i}-{\hat{y}}_{i}\right)}^{2}}{\sum_{i=1}^{n}{\left(y_{i}-\bar{y}\right)}^{2}}$$

(2) $$RMSE=\sqrt{\frac{1}{n}\sum_{i=1}^{n}{\left(y_{i}-{\hat{y}}_{i}\right)}^{2}}$$

(3) $$MAE=\frac{1}{n}\sum_{i=1}^{n}\left|y_{i}-{\hat{y}}_{i}\right|$$

where $y_{i}$ and ${\hat{y}}_{i}$ represent the UAV-LiDAR-derived and model-predicted canopy height values for sample *i*, respectively; $\bar{y}$ denotes the arithmetic mean of the UAV-derived canopy height values; and *n* is the total number of samples.

To interpret the contribution of individual predictors, SHapley Additive exPlanations (SHAP) analysis was applied to the final model using the development dataset (Li et al., 2024a). SHAP values were used to quantify the relative importance and directional effects of the selected remote-sensing variables on canopy-height predictions.

#### 2.2.4 Parametric modeling for AGB estimation

Given the limited size of the field inventory dataset, a parsimonious parametric regression approach was adopted to reduce the risk of overfitting. Forest AGB is closely related to canopy structure through allometric scaling, while optical vegetation indices provide complementary information on canopy spectral characteristics. Accordingly, satellite-retrieved mean canopy height (*H*) was used to characterize vertical stand structure, and vegetation indices (*VIs*) were incorporated as complementary spectral predictors. To match the spatial support of field observations and remote-sensing predictors, the 10-m canopy-height and vegetation-index layers were summarized within each 20 × 20 m field plot, and the resulting plot-level means were paired with field-derived AGB for model calibration and validation. Following the structural-spectral modeling rationale of Yang et al. (2022), an additive log-linear formulation was adopted to separately quantify the contributions of structural and spectral information, as expressed in Equation 4:

(4) $$\ln(AGB)=\beta_{0}+\beta_{1}\times VI+\beta_{2}\times \ln(H)+\epsilon$$

where *AGB* represents plot-level aboveground biomass density (Mg ha^−1^); *H* represents the plot-level satellite-retrieved mean canopy height (m); *VI* denotes the plot-aggregated mean vegetation index, represented by either mean NDVI or mean EVI; $\beta_{0}$ is the intercept; $\beta_{1}$ and $\beta_{2}$ are regression coefficients associated with the spectral and structural predictors, respectively; and *ϵ* represents the residual error term. The logarithmic transformation of *H* reflects the expected scaling relationship between canopy structure and biomass, while the *VI* term accounts for additional spectral variation beyond that explained by *H*.

Predictions on the logarithmic scale were back-transformed to the original AGB scale using a Baskerville bias correction factor given in Equation 5:

(5) $$CF=\exp(RSE^{2}/2)$$

where *RSE* denotes the residual standard error of the fitted model. During cross-validation, the correction factor was estimated independently from the training data in each iteration to avoid information leakage. Two candidate structural-spectral models were constructed using mean NDVI and mean EVI, respectively, with canopy height retained as the structural predictor. Model performance was evaluated using leave-one-out cross-validation (LOOCV) based on R^2^, RMSE, and MAE. The best-performing formulation was subsequently refitted using all field plots to obtain the final coefficients for regional AGB mapping. The stability and uncertainty of the selected structural-spectral model were further assessed using non-parametric bootstrap resampling with 2,000 iterations, from which 95% confidence intervals (CI) were derived for the regression coefficients and LOOCV performance metrics. To assess the complementarity contributions of structural and spectral information, the structural-spectral models were also compared with the corresponding canopy-height-only and vegetation-index-only models under the same LOOCV procedure.

To evaluate whether canopy height provided additional predictive value beyond direct satellite-based AGB estimation, we developed a LASSO regression benchmark using plot-aggregated predictors from Sentinel-1, Sentinel-2, and SDGSAT-1. LASSO was selected because only 32 field plots were available for AGB calibration relative to the large and highly correlated predictor set, allowing simultaneous coefficient shrinkage and variable selection to reduce overfitting. Predictive performance was assessed using nested LOOCV, with λ selected by five-fold cross-validation within each training set. Model performance was evaluated using R^2^, RMSE, MAE, and bias. The overall research design and workflow are summarized in **Figure 3**.

**Figure 3:**
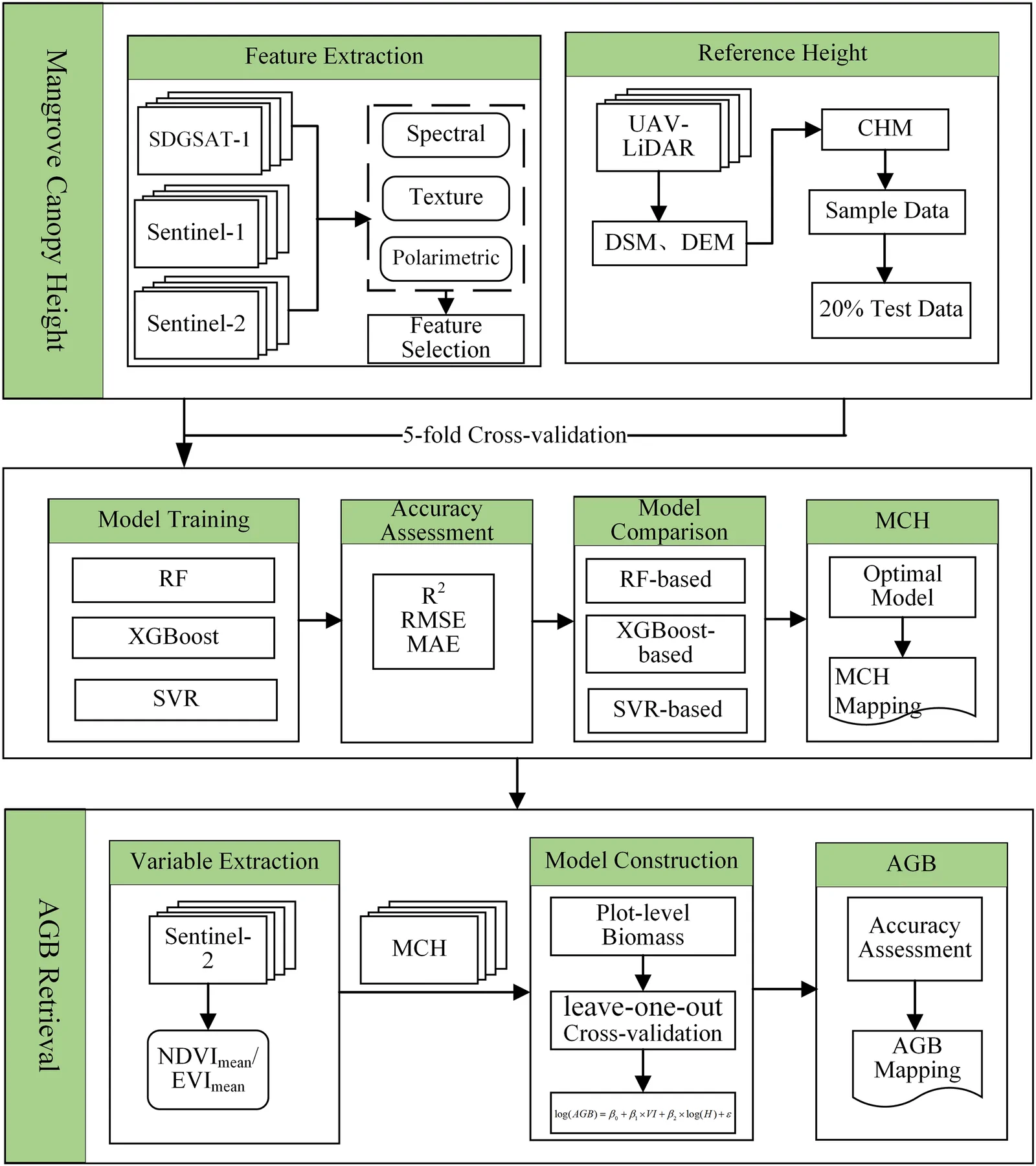
Methodological framework and operational workflow of the study, delineating the sequential stages of remote sensing feature extraction, mangrove canopy height (MCH) modeling and comparison, and final AGB retrieval and spatial mapping.

## 3 Results

### 3.1 Performance evaluation of canopy height inversion models

The observed and predicted canopy height distributions from the three machine-learning regressors showed high consistency, particularly within the dominant 2–5 m range (**Figure 4**). The marginal distributions of canopy height between the development and held-out test datasets were highly comparable, indicating similar canopy height characteristics between the two subsets.

**Figure 4:**
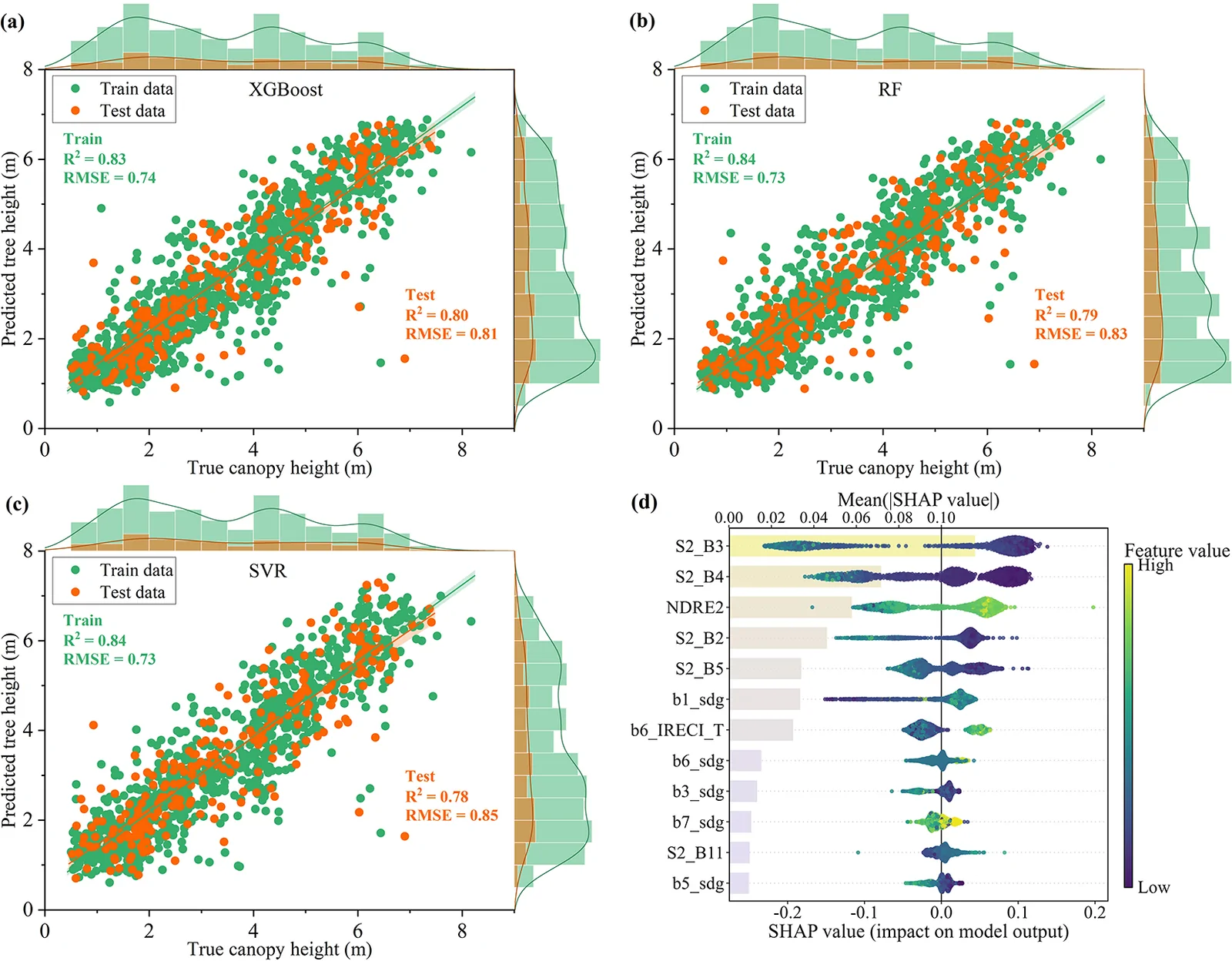
Performance evaluation and SHAP-based interpretation of machine learning models for mangrove canopy height retrieval. Panels **(a–c)** show the scatter plots between observed and predicted canopy heights for the XGBoost, RF, and SVR models, respectively, with corresponding development and held-out test performance metrics (R^2^ and RMSE). Panel **(d)** presents the SHAP beeswarm summary plot illustrating the relative importance and contribution directions of the top 12 remote sensing variables selected from multi-source datasets for canopy height prediction.

Among the candidate models, XGBoost exhibited the highest predictive performance, achieving a test-set R^2^ of 0.80 and an RMSE of 0.81 m. The predicted values were closely aligned with the 1:1 reference line, indicating strong agreement between observed and estimated canopy heights (**Figure 4a**). The RF model showed a slight overfitting tendency; although it achieved high performance on the development subset (R^2^ = 0.84), its performance decreased slightly on the held-out test set (R^2^ = 0.79, RMSE = 0.83 m) (**Figure 4b**). SVR exhibited the lowest predictive performance, with a test-set R^2^ of 0.78 and an RMSE of 0.85 m (**Figure 4c**). Across different canopy height strata, prediction discrepancies were mainly concentrated at the distribution extremes (**Figures 4a–c**). In the high-canopy segment (>6 m), all models showed a slight tendency toward underestimation, with SVR exhibiting the largest deviation from the 1:1 reference line. In contrast, XGBoost more closely reproduced the observed canopy height distribution, including the upper tail. The predictors retained in the final XGBoost model for regional canopy-height mapping are listed in **Supplementary Table S3**.

The SHAP analysis identified S2_B3 as the most influential predictor of mangrove canopy height (**Figure 4d**), followed by S2_B4, NDRE2, S2_B2, and S2_B5. Overall, Sentinel-2 spectral variables were prominent among the highest-ranking predictors, while SDGSAT-1 bands and texture features provided additional contributions. The SHAP distributions further revealed nonlinear and feature-dependent effects on canopy-height predictions.

### 3.2 Spatial heterogeneity of mangrove canopy height across geomorphic settings

The continuous spatial distribution of mangrove canopy height was generated using the optimized XGBoost inversion framework (**Figure 5**). The predicted canopy height exhibited pronounced spatial heterogeneity, ranging from 0.60 to 6.80 m, with a clear core-high and edge-low pattern (**Figures 5a, b**). Mangrove forests were distributed along exposed coastlines, estuarine mudflats, and tidal channels, forming fragmented patches and elongated fringe belts. At the landscape scale, higher canopy heights were concentrated in interior zones and eastern sectors, whereas lower canopy statures were primarily distributed across tidal shoals and marginal ecotones. Across the four representative subregions (**Figures 5a–d**), canopy structural patterns differed markedly among contrasting geomorphic settings.

**Figure 5:**
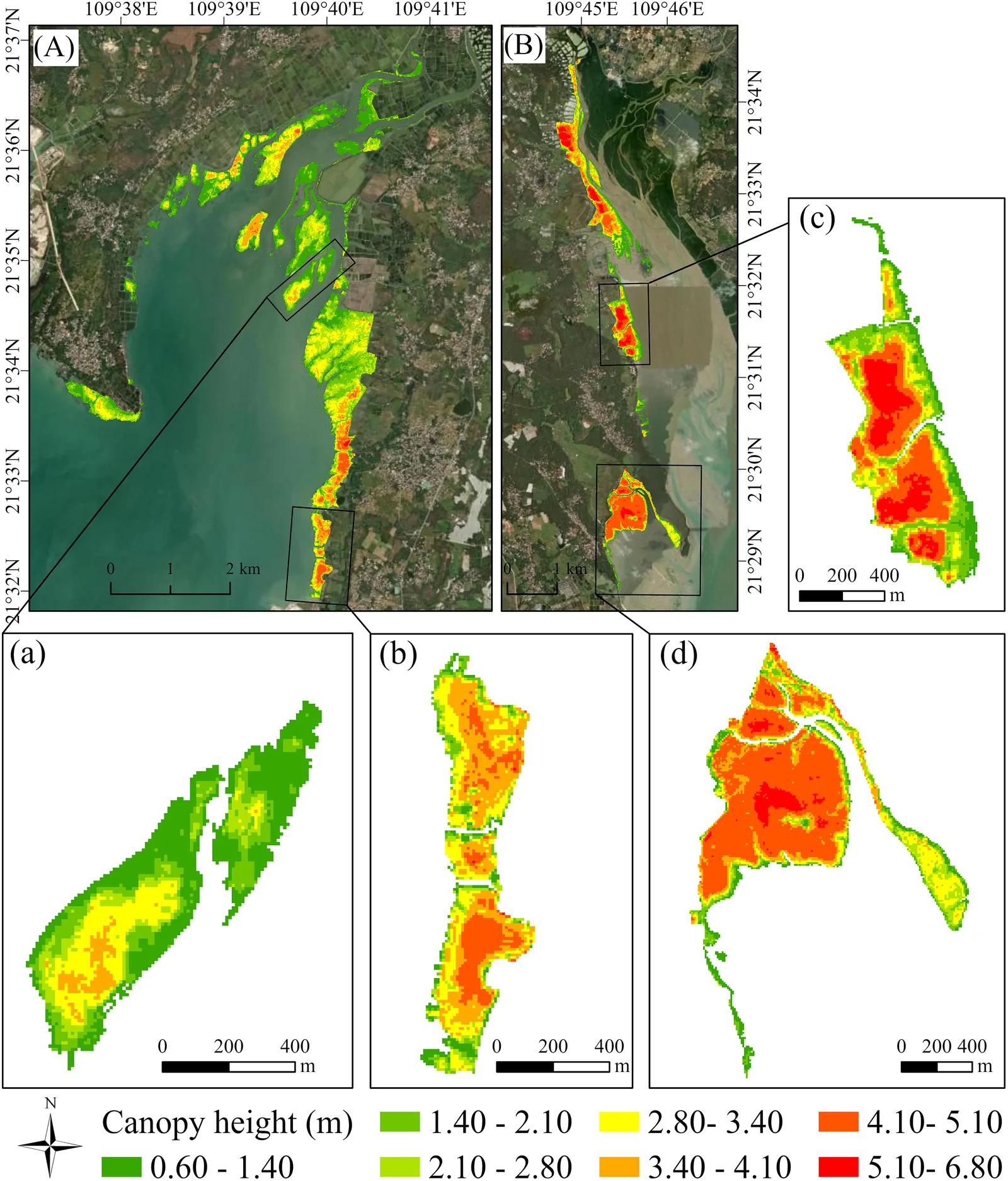
Spatial distribution of mangrove canopy height across different geomorphic settings estimated by the optimized XGBoost model. Panels **(A, B)** provide the regional overview of predicted mangrove canopy height across the study area. Panels **(a–d)** show detailed subsets of four representative regions, illustrating heterogeneous canopy structural patterns associated with contrasting geomorphic and tidal environments.

Within the isolated western tidal shoal of the Dandou Sea, sparse mangrove stands exhibited a relatively narrow canopy height range (0.60–3.40 m), reflecting predominantly low-stature canopy conditions (**Figure 5a**). Along the western tidal channel of the Dandou Sea, the elongated north–south-oriented fringe displayed a pronounced stature gradient (2.10–5.10 m), forming a relatively continuous vegetated corridor (**Figure 5b**). In contrast, the isolated interior mangrove patch in the eastern Xianmi Estuary consisted of highly contiguous tall stands (>4.10 m), reaching a maximum canopy height of 6.80 m and exhibiting a relatively tall and continuous canopy structure (**Figure 5c**). Toward the southern contiguous mangrove forests of Yingluo Bay, canopy height showed a broad medium-to-high gradient, transitioning progressively from low-stature seaward pioneer fringes to taller interior stands (**Figure 5d**).

### 3.3 Structural-spectral AGB modeling and regional mapping

LOOCV showed that the structural-only model based on satellite-retrieved canopy height achieved an R^2^ of 0.63 and an RMSE of 20.23 Mg ha^−1^, outperforming the spectral-only models (**Table 2**). Incorporating vegetation indices further improved model performance, with the EVI-based structural-spectral model achieving the highest predictive accuracy (R^2^ = 0.68, RMSE = 18.72 Mg ha^−1^, MAE = 14.98 Mg ha^−1^). The direct satellite-based LASSO benchmark showed lower predictive performance (R^2^ = 0.48, RMSE = 23.88 Mg ha^-1^), corresponding to an approximately 21.6% lower RMSE for the structural-spectral model. The EVI-based model was therefore selected for regional AGB mapping. To assess the sensitivity of AGB prediction to canopy-height retrieval uncertainty, we further compared two otherwise identical structural-spectral models using UAV-LiDAR-derived and satellite-retrieved canopy height, respectively. Both models used mean EVI as the spectral predictor and were evaluated using the same LOOCV procedure. The two canopy-height estimates showed strong agreement (r = 0.98, RMSE = 0.27 m, MAE = 0.14 m), while the corresponding AGB models achieved RMSEs of 20.66 and 18.72 Mg ha^-1^, respectively. These results suggest that the observed canopy-height retrieval errors did not cause a systematic loss of plot-level AGB predictive performance.

**Table 2:** Leave-one-out cross-validation performance of direct satellite, spectral-only, structural-only, and structural-spectral models for mangrove AGB estimation.

Model type	VI	R^2^	RMSE (Mg ha^-1^)	MAE (Mg ha^-1^)
Structural-spectral	EVI_mean	0.68	18.72	14.98
Structural-spectral	NDVI_mean	0.64	19.76	15.81
Structural-only	—	0.63	20.23	16.68
Spectral-only	NDVI_mean	0.23	29.11	22.64
Spectral-only	EVI_mean	0.06	32.05	25.08
direct satellite	Multi-source satellite predictors	0.48	23.88	19.18

After refitting the selected model using all 32 field plots, the final model yielded an R^2^ of 0.76, an RMSE of 17.05 Mg ha^−1^, and an MAE of 13.57 Mg ha^−1^ (**Figure 6**). Bootstrap analysis further quantified model uncertainty, with confidence intervals for the regression coefficients and LOOCV performance metrics provided in **Supplementary Table S4**. Fitted and observed AGB values generally followed the 1:1 reference line, with some overestimation at low biomass values and underestimation at the upper end of the biomass range. After bias correction for back-transformation, the final model used for regional mapping is expressed in Equation 6.

**Figure 6:**
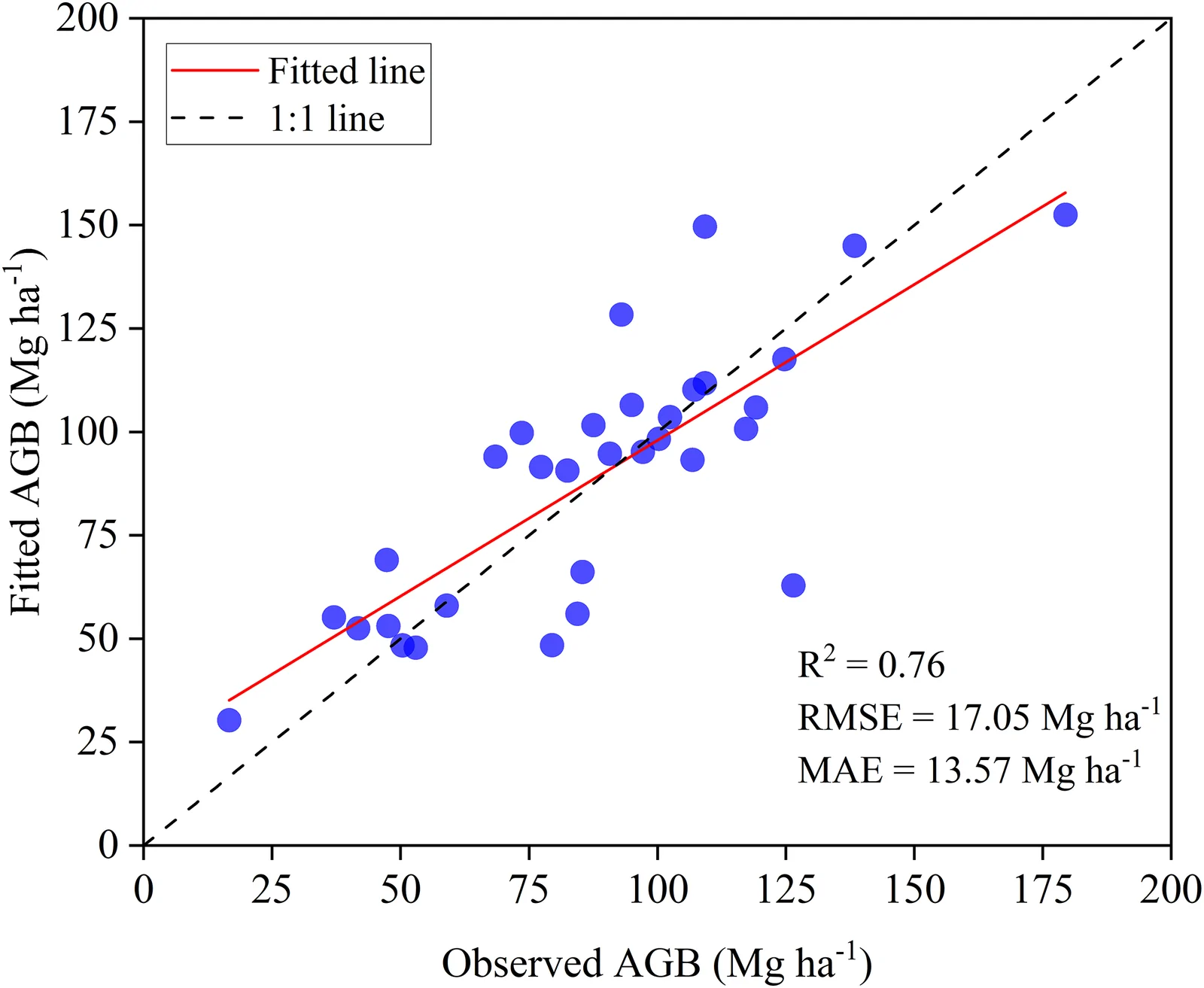
Comparison of field-observed and model-fitted mangrove AGB for the final EVI-based structural-spectral model refitted to all 32 field plots. The black dashed line represents the 1:1 reference line, and the red solid line represents the fitted regression relationship. Reported statistics describe the goodness-of-fit of the final refitted model.

(6) $$AGB=1.03\times \exp(3.19-1.41\times EVI_{mean}+1.47\times \ln(H))$$

The regional AGB map exhibited a spatial pattern broadly consistent with the retrieved canopy-height distribution (**Figures 7A, B**). Pixel-level AGB estimates ranged from 7.9 to 213.4 Mg ha^−1^. High-biomass areas (>105.0 Mg ha^−1^) were mainly concentrated in interior mangrove stands, particularly within the central and eastern sectors of the study area, and generally coincided with taller canopy conditions. In contrast, low-to-moderate AGB values were predominantly distributed along seaward edges and outer mangrove fringes, forming a pronounced edge-to-interior biomass gradient. Overall, total mangrove AGB within the Guangxi Shankou National Mangrove Nature Reserve was estimated at 44,112 Mg, corresponding to a mean density of 49.20 Mg ha^−1^ (**Figure 7C**).

**Figure 7:**
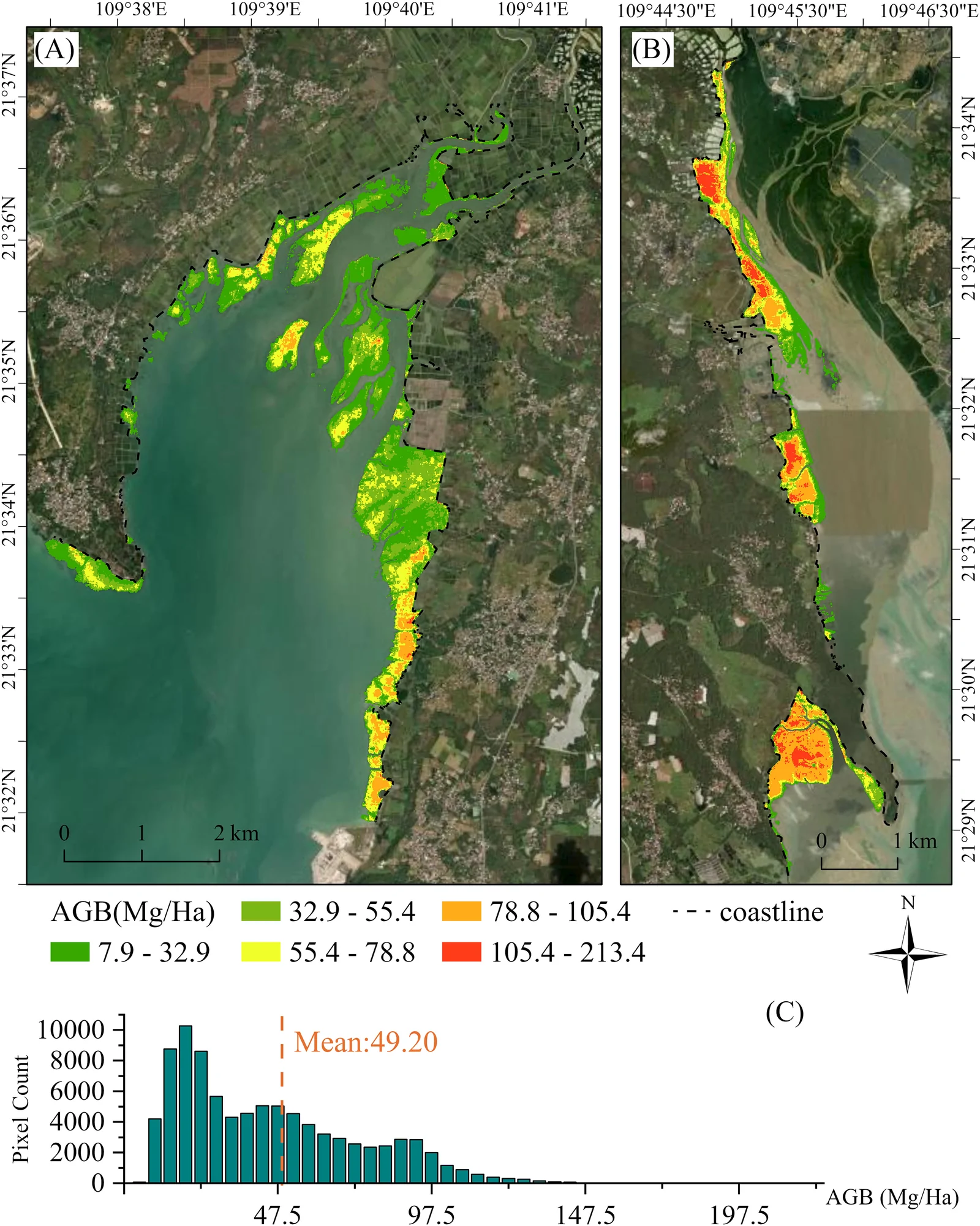
Spatial distribution and heterogeneity of mangrove aboveground biomass (AGB) across the study area. **(A)** Pixel-level AGB distribution in the Dandou Sea sector; **(B)** Pixel-level AGB distribution in the Xianmi Estuary and Yingluo Bay sectors; and **(C)** frequency distribution of pixel-level AGB estimates across the study area.

### 3.4 Spatial trajectories of mangrove AGB across coastal distance gradients

To characterize AGB variation along the coastal-distance gradient, sequential distance zones were generated from the land-coastline baseline and intersected with the mangrove extent of each representative sector. Mean pixel-level AGB was calculated within each distance zone. Distinct spatial patterns were observed with increasing seaward distance from the land coastline across different sectors (**Figure 8**). On the isolated tidal islet in the Dandou Sea, AGB gradually increased with increasing seaward distance from the land coastline, reaching a maximum in the 700–800 m zone (~50 Mg ha^−1^), before declining farther seaward (**Figure 8a**). Along the eastern fringe of the Dandou Sea, the highest biomass occurred within 50–150 m of the land coastline (~73 Mg ha^−1^), followed by a continuous decrease with increasing seaward distance (**Figure 8b**). In contrast, both the Xianmi Estuary (**Figure 8c**) and Yingluo Bay (**Figure 8d**) exhibited unimodal distributions, with peak AGB occurring at intermediate seaward distances (150–200 m and 300–500 m, respectively), followed by gradual declines farther seaward.

**Figure 8:**
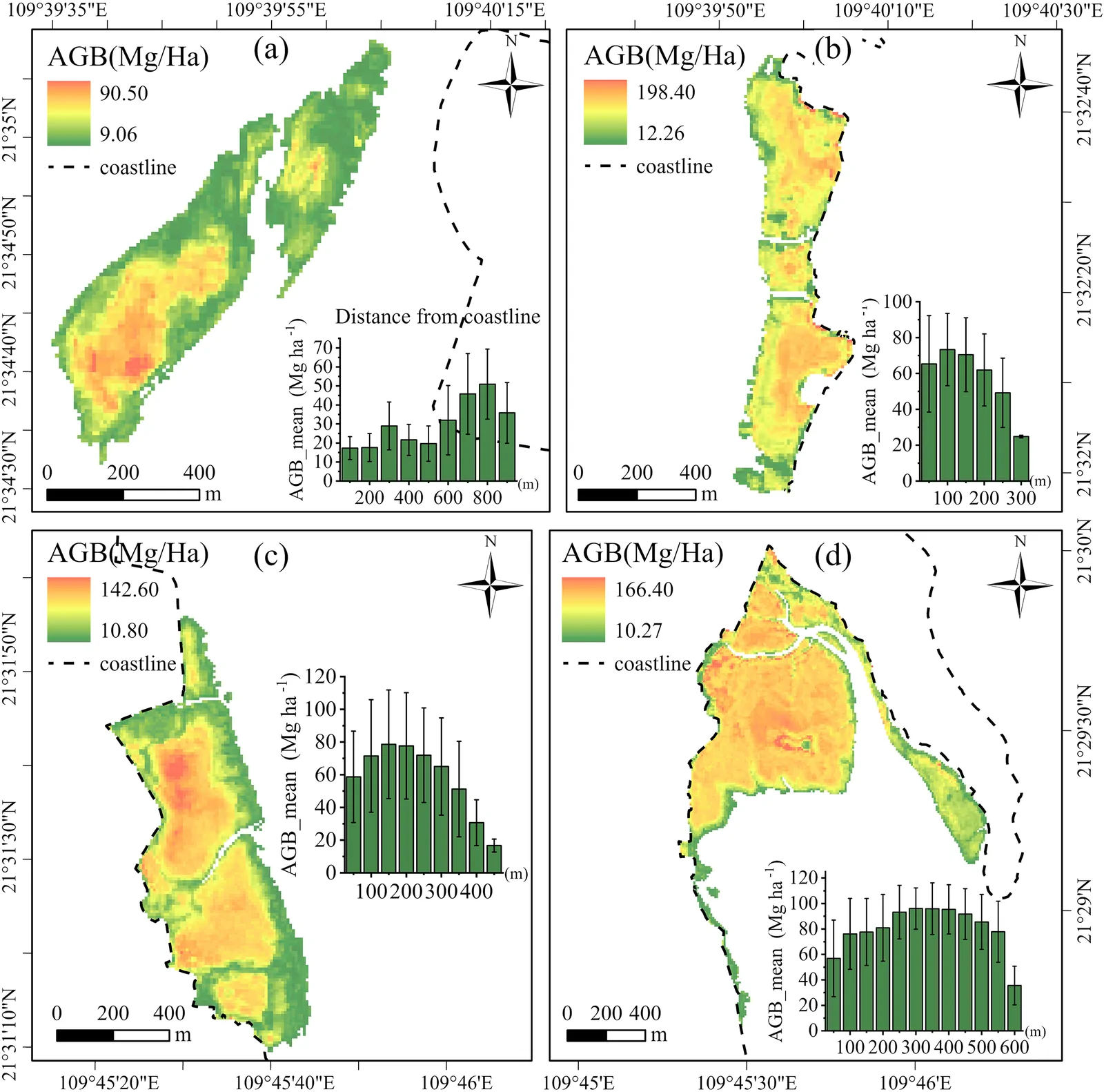
Spatial variations of mangrove AGB along the seaward distance gradient from the land coastline. **(a)** The isolated tidal islet within the Dandou Sea; **(b)** the linear mangrove fringe along the eastern coast of the Dandou Sea; **(c)** the Xianmi Estuary; and **(d)** the interior mangrove patches of Yingluo Bay. The inset bar charts show the mean AGB ± SD within sequential distance buffer zones defined from the coastline baseline. A 100-m interval was used for the isolated tidal islet in the Dandou Sea **(a)**, whereas 50-m intervals were used for the other three sectors **(b–d)**.

## 4 Discussion

### 4.1 Performance of machine learning regressors and sensitivity of predictor matrices

To address the spatial fragmentation and structural heterogeneity of coastal mangrove ecosystems, three machine learning algorithms (RF, XGBoost, and SVR) were compared for canopy height inversion. Their ability to capture nonlinear relationships between multi-source remote sensing features and forest structural attributes provides an effective approach for quantifying mangrove vertical structure across complex coastal environments.

Among the three models, XGBoost achieved the best predictive performance on the held-out test set (R^2^ = 0.80). This performance was within the range reported in previous studies. For example, an RF model integrating multi-source satellite and UAV-LiDAR data achieved an R^2^ of 0.71 (Chen et al., 2023b), while least-squares support vector machines reached R^2^ values of up to 0.82 across different forest structural conditions (García et al., 2018). However, RF and SVR exhibited a tendency toward underestimation in tall-canopy plots (>6 m), where predicted values converged toward the sample mean, indicating regression-to-the-mean effects. This bias likely resulted from the limited representation of tall canopy conditions in the development subset, which constrained model capability in capturing extreme structural values. Accurate vegetation-height estimation also depends on the quality of structural observations, and improved UAV point-cloud processing has been shown to enhance height retrieval accuracy (Feng et al., 2026).

SHAP analysis further indicated that Sentinel-2 predictors contributed more strongly to the fitted model, possibly because of their sensitivity to canopy spectral properties and the greater signal stability associated with multi-temporal compositing (Upakankaew et al., 2022). SDGSAT-1 provided complementary information through its 10-m multispectral observations, whereas Sentinel-1 SAR variables showed relatively lower importance. This may reflect the comparatively limited sensitivity of C-band backscatter to canopy-height variation in dense mangrove stands, as well as its susceptibility to tidal and substrate-related effects (Pradisty et al., 2025).

### 4.2 Methodological synergies between predicted canopy height and spectral indices for biomass mapping

Canopy height represents a key descriptor of vertical structural complexity in mangrove ecosystems and provides an essential structural basis for AGB estimation (Tamiminia et al., 2024). In this study, the retrieved canopy height products revealed pronounced spatial heterogeneity, with taller stands concentrated in interior zones and shorter canopies distributed along seaward margins and intertidal transition areas, consistent with previous field-based observations (Potapov et al., 2021; Lang et al., 2023; Jia et al., 2025). Although UAV-LiDAR enables highly accurate structural characterization, its application for regional-scale biomass assessment remains constrained by limited spatial coverage and dependence on localized acquisitions (Cao et al., 2019; Tian et al., 2021; Pan et al., 2023). Integrating multi-source satellite observations for canopy height retrieval provides an effective means of extending localized structural information across heterogeneous landscapes while reducing reliance on extensive LiDAR acquisitions. More importantly, satellite-retrieved canopy height provides a physically interpretable structural link between localized UAV-LiDAR observations and regional AGB estimation, allowing vertical stand structure to be explicitly represented in the biomass model.

Beyond structural parameters, multispectral vegetation indices provide complementary spectral information related to canopy closure, vegetation condition, and localized canopy variability (Deng et al., 2026). In this study, the structural-only model based on satellite-retrieved canopy height substantially outperformed the spectral-only models, while the addition of mean EVI further improved predictive accuracy, indicating that spectral information provides complementary predictive value beyond canopy height alone. The direct satellite-based LASSO benchmark also showed lower predictive performance than the structural-spectral model (R^2^ = 0.48, RMSE = 23.88 Mg ha^−1^ vs. R^2^ = 0.68, RMSE = 18.72 Mg ha^−1^, further indicating that canopy height provides structural information not fully captured by the satellite predictors alone. Highly flexible models such as RF, XGBoost, and RBF-SVR were not prioritized for direct AGB estimation because, under limited training data, their greater model complexity and hyperparameter-tuning requirements may increase the risk of overfitting and unstable generalization. By integrating satellite-retrieved canopy height with multispectral vegetation indices within a structural-spectral log-linear model, the framework supported regional AGB estimation from limited field observations. The estimated total mangrove AGB within the Shankou National Mangrove Nature Reserve reached 44,112 Mg, with a mean density of 49.20 Mg ha^-1^, which was within the range reported in previous studies (Ji et al., 2026; Liu et al., 2024a). Overall, this structural-spectral framework provides a scalable approach for regional mangrove biomass assessment and blue carbon management.

### 4.3 Methodological constraints and technological outlook for mangrove AGB retrieval

Two primary limitations may affect the uncertainty and applicability of the current framework. First, the lack of species-level classification may overlook intra-community heterogeneity in canopy morphology, structural architecture, and species-specific differences in biomass–structure relationships (Li et al., 2024b; Fu et al., 2025). Without explicit taxonomic stratification, the model may have reduced ability to distinguish different spectral-structural relationships among mangrove communities, thereby introducing additional uncertainty into biomass estimation. Second, prediction errors remained at the upper ends of both canopy height and biomass distributions. The canopy-height models showed some underestimation for tall stands, which may propagate into regional AGB estimates. In addition, optical vegetation indices generally become less sensitive as canopy closure and biomass increase (Huete et al., 1997; Mutanga et al., 2023), potentially limiting their contribution in high-biomass stands. Although the stratified random sampling strategy improved the representation of structural gradients, some prediction compression and regression-to-the-mean effects remained in high-biomass regions.

Future studies should further investigate multi-sensor fusion strategies to improve prediction in high-biomass stands. Integrating optical observations with multi-polarized SAR backscatter and GEDI spaceborne LiDAR waveform information may provide complementary structural constraints and improve regional biomass estimation. Additionally, the empirical biomass model could be improved by incorporating additional canopy structural attributes, including leaf area index (Xing et al., 2024) and related metrics. UAV LiDAR-derived metrics, such as canopy cover, canopy density, and other descriptors of vertical forest structure, may capture variations in forest architecture and biomass allocation more comprehensively than canopy height alone, thereby reducing prediction uncertainty, particularly in mature and structurally complex mangrove stands. Integrating these complementary structural and spectral observations may therefore improve the accuracy, robustness, and transferability of regional biomass estimation.

## 5 Conclusions

This study developed a hierarchical framework for regional mangrove AGB mapping by extending localized UAV-LiDAR structural observations through multi-source satellite data. The framework uses satellite-retrieved canopy height as a structural bridge and integrates it with spectral information within a structural-spectral log-linear model. Cross-validated comparisons showed that the canopy-height-only model substantially outperformed the spectral-only models, while the inclusion of mean EVI yielded a modest but consistent improvement over the structural-only model, supporting the complementary value of structural and spectral information for AGB estimation. The resulting 10-m AGB map revealed pronounced spatial heterogeneity across the Shankou National Mangrove Nature Reserve, with higher biomass generally concentrated in interior stands and lower biomass along seaward margins. Biomass trajectories also differed among contrasting geomorphic settings, suggesting that mangrove structural development varies across coastal environmental gradients. Overall, this framework provides a practical and scalable approach for regional mangrove biomass assessment while reducing reliance on wall-to-wall active sensing, with potential applications in blue carbon accounting, coastal wetland conservation, and adaptive ecosystem management.

## Data Availability

The original contributions presented in the study are included in the article/**Supplementary Material**, further inquiries can be directed to the corresponding author/s.
