# Supplementary material for: From plot to region: canopy height-mediated mangrove aboveground biomass mapping by synergizing UAV-LiDAR and multi-source satellite data
Source: Front Plant Sci. 2026 Oct 9;17:1964480. doi: 10.3389/fpls.2026.1964480 (PMC13691593; doi:10.3389/fpls.2026.1964480)
Supplement: Supplementary file 1 [file Datasheet1.docx]

**Supplementary Figure**

Figure S1. Spatial distribution of the development and held-out test samples used for canopy-height modeling. UAV flight strips, mangrove extent, and the study-area boundary are also shown for spatial reference.

**Supplementary Table**

Table S1. Summary of multi-source remote sensing feature variables derived from Sentinel-1/2 and SDGSAT-1 imagery for canopy height estimation.

| **Sensor** | **Feature Description** | **Abbreviation** | **Formulation** |
| --- | --- | --- | --- |
| **Sentinel-1 SAR** | Vertical-Vertical Polarization | VV | - |
|  | Vertical-Horizontal Polarization | VH | - |
|  | Radar Index | RI | VH / VV |
|  | Normalized Difference Index | NDI | (VV - VH) / (VV + VH) |
|  | Radar Vegetation Index | RVI | (4 × VH) / (VV + VH) |
| **Sentinel-2 MSI** | Band 2-Blue (460-525 nm) | S2_B2 | - |
|  | Band 3-Green (542-577 nm) | S2_B3 | - |
|  | Band 4-Red (650-680 nm) | S2_B4 | - |
|  | Band 5-Red Edge1 (695-709 nm) | S2_B5 | - |
|  | Band 6-Red Edge2 (733-747 nm) | S2_B6 | - |
|  | Band 7-Red Edge3 (768-796 nm) | S2_B7 | - |
|  | Band 8-NIR (780-885 nm) | S2_B8 | - |
|  | Band 8A -Narrow NIR (855-876 nm) | S2_B8A | - |
|  | Band 11-SWIR1 (1569-1659 nm) | S2_B11 | - |
|  | Band 12-SWIR2 (2115-2289 nm) | S2_B12 | - |
|  | Normalized Difference Vegetation Index | NDVI | (B8 - B4) / (B8 + B4) |
|  | Enhanced Vegetation Index | EVI | 2.5 × (B8 - B4) / (B8 + 6 × B4 - 7.5 × B2 + 1) |
|  | Normalized Difference Infrared Index 1 | NDII1 | (B8A - B11) / (B8A + B11) |
|  | Normalized Difference Infrared Index 2 | NDII2 | (B8A - B12) / (B8A + B12) |
|  | Normalized Difference Red Edge Index | NDRE | (B8 - B5) / (B8 + B5) |
|  | Normalized Difference Red Edge Index 1 | NDRE1 | (B8A - B5) / (B8A + B5) |
|  | Normalized Difference Red Edge Index 2 | NDRE2 | (B8A - B6) / (B8A + B6) |
|  | Index of Reflectance Enhanced Crop Index | IRECI | ((B7 - B4) × B6) / B5 |
|  | Normalized Difference Shortwave Infrared Index | NDSI | (B11 - B12) / (B11 + B12) |
|  | Normalized Difference Water Index | NDWI | (B3 - B8) / (B3 + B8) |
|  | Normalized Difference Built-up Index | NDBI | (B8 - B12) / (B8 + B12) |
|  | Modified Soil-Adjusted Vegetation Index | MSAVI | ((2 × B8 + 1 – sqrt ((2 × B8 + 1) ² - 8 × (B8 - B4))) / 2 |
|  | Difference Vegetation Index | DVI | B8 - B4 |
|  | Enhanced Built-up Soil Index | EBSI | 2 × B8 - B11 - B4 - 0.5 |
|  | Land Surface Water Index | LSWI | (B8 - B11) / (B8 + B11) |
|  | Soil-Adjusted Vegetation Index | SAVI | (B8 - B4) / (B8 + B4 + 0.5) × 1.5 |
|  | Reflectance Enhanced 75 Index | RE_75 | (B7 - B5) / (B7 + B5) |
|  | Near Infrared to Red Edge Ratio | NIR_RE | B8A / B5 |
|  | contrast | b1_BN_T | - |
|  | (B4/ B8/ B8A/ B11/ NDVI/ IRECI) |  |  |
|  | entropy | b2_BN_T | - |
|  | (B4/ B8/ B8A/ B11/ NDVI/ IRECI) |  |  |
|  | Homogeneity (B4/ B8/ B8A/ B11/ NDVI/ IRECI) | b3_BN_T | - |
|  | second_moment (B4/ B8/ B8A/ B11/ NDVI/ IRECI) | b4_BN_T | - |
|  | Correlation (B4/ B8/ B8A/ B11/ NDVI/ IRECI) | b5_BN_T | - |
|  | Variance (B4/ B8/ B8A/ B11/ NDVI/ IRECI) | b6_BN_T | - |
|  | Dissimilarity (B4/ B8/ B8A/ B11/ NDVI/ IRECI) | b7_BN_T | - |
| **SDGSAT-1 MSI** | Band 1-Deep blue (374-427 nm) | b1_sdg | - |
|  | Band 2-Deep blue (410-467 nm) | b2_sdg | - |
|  | Band 3-blue (457-529 nm) | b3_sdg | - |
|  | Band 4-Green (510-597 nm) | b4_sdg | - |
|  | Band 5-Red (618-696 nm) | b5_sdg | - |
|  | Band 6-Red edge (744-813 nm) | b6_sdg | - |
|  | Band 7-Near infrared (798-911 nm) | b7_sdg | - |

Table S2. Hyperparameter search ranges and optimal settings for the XGBoost, RF, and SVR models.

| **Model** | **Hyperparameter** | **Search range** | **Optimal value** |
| --- | --- | --- | --- |
| XGBoost | n_estimators | 200, 500 | 200 |
|  | max_depth | 3, 4, 5 | 4 |
|  | learning_rate | 0.03, 0.05, 0.10 | 0.05 |
|  | subsample | 0.8, 1.0 | 0.8 |
|  | colsample_bytree | 0.8, 1.0 | 0.8 |
|  | reg_alpha | 0, 0.1 | 0 |
|  | reg_lambda | 1, 2 | 1 |
| RF | n_estimators | 200, 500, 800 | 200 |
|  | max_depth | None, 5, 10, 20 | 10 |
|  | min_samples_split | 2, 5, 10 | 2 |
|  | min_samples_leaf | 1, 2, 4 | 1 |
|  | max_features | sqrt, log2, None | None |
| SVR | kernel | rbf, linear | rbf |
|  | C | 0.1, 1, 10, 100 | 1 |
|  | epsilon | 0.01, 0.05, 0.1, 0.5 | 0.1 |
|  | gamma | scale, auto | scale |

Table S3. Final predictor set used in the XGBoost model for regional canopy-height mapping.

| **Sensor** | **Feature Description** | **Abbreviation** |
| --- | --- | --- |
| **Sentinel-2 MSI** | Band 2-Blue (460-525 nm) | S2_B2 |
|  | Band 3-Green (542-577 nm) | S2_B3 |
|  | Band 4-Red (650-680 nm) | S2_B4 |
|  | Band 5-Red Edge1 (695-709 nm) | S2_B5 |
|  | Band 6-Red Edge2 (733-747 nm) | S2_B6 |
|  | Band 7-Red Edge3 (768-796 nm) | S2_B7 |
|  | Band 8-NIR (780-885 nm) | S2_B8 |
|  | Band 8A -Narrow NIR (855-876 nm) | S2_B8A |
|  | Band 11-SWIR1 (1569-1659 nm) | S2_B11 |
|  | Band 12-SWIR2 (2115-2289 nm) | S2_B12 |
|  | Normalized Difference Vegetation Index | NDVI |
|  | Enhanced Vegetation Index | EVI |
|  | Normalized Difference Infrared Index 1 | NDII1 |
|  | Normalized Difference Infrared Index 2 | NDII2 |
|  | Normalized Difference Red Edge Index | NDRE |
|  | Normalized Difference Red Edge Index 1 | NDRE1 |
|  | Normalized Difference Red Edge Index 2 | NDRE2 |
|  | Index of Reflectance Enhanced Crop Index | IRECI |
|  | Normalized Difference Shortwave Infrared Index | NDSI |
|  | Normalized Difference Water Index | NDWI |
|  | Normalized Difference Built-up Index | NDBI |
|  | Modified Soil-Adjusted Vegetation Index | MSAVI |
|  | Difference Vegetation Index | DVI |
|  | Enhanced Built-up Soil Index | EBSI |
|  | Land Surface Water Index | LSWI |
|  | Soil-Adjusted Vegetation Index | SAVI |
|  | Reflectance Enhanced 75 Index | RE_75 |
|  | Near Infrared to Red Edge Ratio | NIR_RE |
|  | contrast | b1_BN_T |
|  | (B4/ B8/ B8A/ B11/ NDVI) |  |
|  | entropy | b2_BN_T |
|  | (B4/ B8/ B8A/ B11/ NDVI/ IRECI) |  |
|  | Homogeneity (B8/ NDVI) | b3_BN_T |
|  | second_moment (B4/ B11/ NDVI) | b4_BN_T |
|  | Correlation (B4/ B11) | b5_BN_T |
|  | Variance (B4/ B8/ B8A/ B11/ NDVI/ IRECI) | b6_BN_T |
|  | Dissimilarity (B4/ B8/ B8A/ B11/ NDVI) | b7_BN_T |
| **SDGSAT-1 MSI** | Band 1-Deep blue (374-427 nm) | b1_sdg |
|  | Band 2-Deep blue (410-467 nm) | b2_sdg |
|  | Band 3-blue (457-529 nm) | b3_sdg |
|  | Band 4-Green (510-597 nm) | b4_sdg |
|  | Band 5-Red (618-696 nm) | b5_sdg |
|  | Band 6-Red edge (744-813 nm) | b6_sdg |
|  | Band 7-Near infrared (798-911 nm) | b7_sdg |

Table S4. Bootstrap uncertainty of regression coefficients and LOOCV performance metrics for the final structural-spectral AGB model.

| **Metric** | **Estimate** | **95% bootstrap CI** |
| --- | --- | --- |
| Intercept | 3.194 | 2.377–3.871 |
| EVI_mean | −1.408 | −3.113–0.505 |
| log(H) | 1.466 | 0.978–1.940 |
| LOOCV R^2^ | 0.681 | 0.341–0.832 |
| RMSE (Mg ha⁻¹) | 18.72 | 14.81–22.33 |
| MAE (Mg ha⁻¹) | 14.98 | 11.24–18.86 |
| Bias (Mg ha⁻¹) | 0.87 | −5.49–7.68 |
